# Anticholinergic Burden in Elderly People in Nursing Homes: Cross-Sectional Assessment Using ACB Calculator and CRIDECO Anticholinergic Load Scale

**DOI:** 10.3390/medicines13020014

**Published:** 2026-04-07

**Authors:** Tânia Nascimento, Maria Ana Matos, Ezequiel Pinto

**Affiliations:** 1 Escola Superior de Saúde da Universidade do Algarve (ESSUAlg), Campus de Gambelas, Edifício 1, 8005-139 Faro, Portugal; a76650@ualg.pt; 2 Algarve Biomedical Center Research Institute (ABC-RI), Campus de Gambelas, Universidade do Algarve, Edifício 2, 8005-139 Faro, Portugal

**Keywords:** anticholinergic burden, ACB calculator, CRIDECO anticholinergic load scale, elderly, nursing homes

## Abstract

Background/Objectives: Anticholinergic burden is an important risk marker in older adults, associated with cognitive decline, falls, and increased mortality. This study aimed to assess anticholinergic burden in institutionalized elderly individuals using two tools (ACB calculator and CALS—CRIDECO Anticholinergic Load Scale), as well as to analyze its relationship with pharmacotherapeutic variables like polypharmacy. Methods: A descriptive cross-sectional study was conducted by analyzing the pharmacotherapeutic profiles of institutionalized elderly individuals (≥65 years) utilizing individualized medication preparation services from a community pharmacy in Alentejo (Portugal). Participants agreed to the study and had complete, up-to-date pharmacotherapeutic profiles. Results: The pharmacotherapeutic profiles of 75 institutionalized elderly people were analyzed; the sample comprised mostly women (72%) who had experienced excessive polypharmacy (≥10 medications) (56%) and had an average age of 85.62 ± 7.62 years. It was found that 90.7% (ACB) and 89.3% (CALS—CRIDECO) of the elderly had anticholinergic burden, with mean values of 3.60 ± 2.84 and 3.33 ± 2.51, respectively. Women exhibited higher anticholinergic burden in unadjusted analyses (*p* < 0.05). The burden correlated moderately with the total number of medications (*p* < 0.05). Conclusions: The results show high exposure to anticholinergic medications in the institutionalized elderly population, reinforcing the rationale for systematic therapeutic reviews focused on the pharmacological safety of institutionalized older adults in community pharmacies.

## 1. Introduction

Population ageing constitutes one of the greatest public health challenges, given the increase in average life expectancy and the rising prevalence of chronic diseases associated with functional dependence [1]. This process is mostly associated with physiological and pharmacokinetic changes that affect the body’s response to medications, thus making the elderly more susceptible to adverse reactions. The presence of polypharmacy, associated with physical and cognitive frailty, contributes to health deterioration and loss of quality of life [2]. Polypharmacy is defined as the simultaneous use of five or more medications [3] and is highly prevalent in the elderly population, especially in institutional settings [4,5,6]. This practice, often a marker of multiple chronic comorbidities rather than an independent cause of harm, is associated with various risks, including drug interactions, an increase in anticholinergic burden, adverse drug reactions, functional and cognitive decline, a higher risk of falls, hospitalizations, and increased mortality. Furthermore, there is a high prevalence of potentially inappropriate medications which, in many cases, could be replaced by safer therapeutic alternatives [7,8,9,10,11].

Anticholinergic drugs primarily act by blocking acetylcholine (muscarinic) receptors, inhibiting its action in the central and peripheral nervous system. These medications can be used in the treatment of various pathologies [12]. Their pharmacodynamics involves the antagonism of cholinergic acetylcholine receptors, more specifically muscarinic receptors. Consequently, the inhibition of normal cholinergic responses in various tissues occurs, leading to situations such as cognitive impairment, sedation, and delirium (central anticholinergic effects), or a reduction in salivary secretions, decreased gastrointestinal motility, or increased heart rate (peripheral anticholinergic effects) [12,13,14]. Anticholinergic drugs can also be associated with a higher risk of falls in the elderly due to muscle weakness, postural instability, and motor incoordination [11,15]. The anticholinergic burden, defined as the cumulative effect of all medications with anticholinergic activity in use, is even more important in the elderly, as they present not only an increase in blood–brain barrier permeability but also a decrease in cholinergic reserves and age-related structural changes in muscarinic receptor binding sites that can negatively impact anticholinergic burden [16,17,18]. The exposure of older adults to drugs individually classified as having a low anticholinergic burden is high, contributing to an elevated overall anticholinergic burden and serious long-term consequences, particularly in institutionalized older adults. Importantly, even medications with individually low anticholinergic activity can contribute substantially to the total burden when prescribed simultaneously, as evidenced by studies demonstrating clinically significant cumulative effects in older adults receiving multiple low-potency anticholinergic agents [19,20,21]. There are several tools to quantify anticholinergic burden, ranging from more “traditional” scales to emerging artificial intelligence models, although none is currently considered the gold standard, which limits direct comparability of anticholinergic burden estimates across studies using different instruments [17].

Cognitive and functional decline are predictive factors for the institutionalization of the elderly. The need for constant support leads to the search for social support structures that allow for closer monitoring of the elderly [22,23]. However, institutionalization can negatively impact these individuals, accelerating functional and cognitive decline, with greater dependence, and exacerbating situations of depression and social isolation, thereby decreasing the quality of life of these residents [24,25]. Polypharmacy and the use of potentially inappropriate medications, namely psychotropics, further enhance the anticholinergic burden and negatively impact the lives of the elderly [26,27]. In this scenario, a multidisciplinary approach focused on the systematic and individualized review of pharmacotherapy becomes essential, respecting the functionality and expectations of each elderly person.

This study had two main objectives: (i) a primary objective of estimating the prevalence and magnitude of anticholinergic burden in institutionalized elderly individuals using two assessment tools—the ACB calculator and the CRIDECO Anticholinergic Load Scale (CALS); and (ii) a secondary, exploratory objective of examining associations between anticholinergic burden and pharmacotherapeutic variables, including polypharmacy, medication regimen complexity, potentially inappropriate medications, and potential drug–drug interactions.

It is important to note that anticholinergic burden, as assessed in cross-sectional and observational studies, functions as a risk marker (a measurable indicator statistically associated with adverse outcomes) rather than an established causal risk factor with a demonstrated independent mechanistic role. Given the cross-sectional design, all analyses are associative and no causal inferences are intended.

## 2. Materials and Methods

A descriptive, cross-sectional study was conducted based on the analysis of pharmacotherapeutic profiles of institutionalized elderly individuals monitored by a community pharmacy providing a multi-dose packaging service. The reference population included 246 institutionalized patients. The final sample consisted of 75 individuals selected through simple random sampling from those who met the following inclusion criteria: age ≥ 65 years, consent to participate, a complete and updated therapeutic profile in the computer system, and the use of at least two medications. Patients with incomplete data or outdated profiles were excluded.

The target sample size was initially estimated at 150 participants, based on a margin of error of 5% for prevalence estimates at the 95% confidence level (reference population *n* = 246). However, practical constraints during data collection—including difficulty obtaining informed consent and incomplete pharmacotherapeutic profiles for a proportion of eligible patients—limited the final sample to 75 individuals. A post hoc precision analysis indicates that this sample size yields a margin of error of approximately 10% for proportions estimated at the 95% confidence level, which is considered acceptable for the descriptive nature of this study.

Data collection started on April 2025 and lasted until October 2025. Informed consent was obtained from the institutions’ staff specifically for this study. After consent was obtained, the data were pseudonymized, with a code assigned to each patient to ensure data privacy.

Data were collected from the pharmacy’s electronic records, including age, sex, a complete list of medications (by International Nonproprietary Name), including non-prescription drugs and dietary supplements and usual dosage. Medication coding was performed according to the Anatomical Therapeutic Chemical (ATC) classification, using the third level to group medications by pharmacological groups [28]. Food supplements or other products were classified as “No ATC.”. The total number of drugs allowed for the classification of patients as non-polypharmacy (<5 medications), polypharmacy (5–9), or excessive polypharmacy (≥10) [3].

A Type 1 medication review was performed according to the Pharmaceutical Care Network Europe (PCNE) classification, using only available clinical and pharmacotherapeutic data, without direct contact with patients or other healthcare professionals [29]. This review allowed for the identification of potential medication-related problems (MRPs), including potential drug–drug interactions and potentially inappropriate medications (PIMs), as well as the assessment of the overall anticholinergic burden.

The anticholinergic burden was calculated using two tools: (i) the ACB calculator [30], a digital platform that combines scores from two different scales, the Anticholinergic Cognitive Burden (ACB) scale and the German Anticholinergic Burden Scale (GABS). This calculator assigns scores from 0 (no burden) to 3 (high burden) to each drug, based on evidence of anticholinergic and cognitive effects, and presents the final sum as the total anticholinergic burden; and (ii) the CRIDECO Anticholinergic Load Scale (CALS—CRIDECO) [31], which classifies medications into low, medium, or high-burden categories, similarly scored from 1 to 3. The total anticholinergic burden per patient results from the sum of the values assigned to each medication, additionally considering the proportion of patients with a total burden ≥ 3, which is frequently used as a higher-risk threshold [31]. The choice of both tools focused on the use of an updated and comprehensive tool (CALS—CRIDECO) developed in Europe, specifically in Spain [31], a country close to Portugal with similar sociodemographic characteristics, and on the use of a digital tool that is accessible, intuitive, and easy to use, as the calculation is automated [30]. In addition, this digital tool integrates two scales (ACB and GABS) that are recognized and widely used in scientific literature [18,32,33]. Although the ACB scale focuses more on the cognitive effects of drugs, GABS is a tool focused on the elderly and more comprehensive in terms of anticholinergic effects [33], as is CALS—CRIDECO [31]. Thus, it was considered that the digital tool could provide a more comprehensive perspective on the results obtained. It should be noted that the precise algorithm used to handle discrepancies between the two integrated scales, for instance, when a drug is classified differently by ACB and GABS, or when it appears in only one of the two lists, is not publicly documented by the platform developers. This represents a transparency limitation inherent to the use of this digital tool, which may affect the reproducibility of individual drug scores.

Potential drug–drug interactions were also evaluated using the Lexicomp (Lexidrug, UpToDate) database, which reflects the theoretical risk of interactions, and classified into categories A (no known interaction), B (no action needed), C (monitor therapy), D (consider therapy modification), and X (avoid combination) [34]. Potentially inappropriate medications were identified based on the European EU(7)-PIM list, operationalized for Portugal using the ApiMedOlder platform [35]. Therapeutic complexity was assessed using the Medication Regimen Complexity Index (MRCI) [36]. The MRCI score is calculated from three subtotals that contribute to the overall complexity. Subtotal A refers to the pharmaceutical form of the medication (e.g., tablet, injection, inhaler), as different forms may have different complexities. Subtotal B refers to the frequency of dosing, considering that more frequent dosing schedules increase complexity. Subtotal C refers to additional instructions and precautions for each drug, such as dietary restrictions or specific administration times. The MRCI score corresponds to the sum of the subtotals. Although no specific MRCI threshold has been validated for institutionalized older adults, the cutoff of >16.5, originally defined for community-dwelling elderly, was applied in the absence of a more appropriate reference value [36,37].

Statistical analysis was performed using IBM SPSS Statistics 29.0, Armonk, NY, USA, applying descriptive statistics (mean, median, standard deviation, interquartile range, and frequencies) and inferential statistics, depending on the distribution of variables as assessed by the Shapiro–Wilk test. For comparisons between categorical groups, the chi-square test was used, while the comparison of metric variables between two groups utilized Student’s *t*-test (parametric data) or Mann–Whitney test (non-parametric data). For three or more groups, Kruskal–Wallis tests were applied, due to non-normality of variables, with post hoc pairwise comparisons using Dunn correction where significant differences were identified. Correlations between anticholinergic burden, number of medications, MRCI, interactions, and PIMs were evaluated using Pearson or Spearman coefficients. The significance level was set at *p* < 0.05.

## 3. Results

Seventy-five institutionalized patients aged 65 years or older were included. The sample was predominantly female (72%; n = 54) and with a mean age of 85.62 ± 7.62 years (median = 86, IQR = 9). No significant age differences were found between sexes (U = 662.5; *p* = 0.203). Approximately 67% (n = 50) were aged 85 years or older.

The total number of medications consumed per individual ranged from 2 to 18, with a mean of 9.95 ± 3.59. Regarding polypharmacy, 38.7% (n = 29) were considered polymedicated (5–9 medications) and 56.0% (n = 42) were classified as having excessive polypharmacy (≥10 medications). Among those with polypharmacy (≥5 medications), 73.2% (n = 52) were female, although there were no statistically significant differences between the sexes (*p* = 0.341).

Regarding the pharmacotherapeutic profile, the most frequently used medications belonged to the nervous system (ATC N), with a mean of 2.95 ± 1.76 drugs per user, followed by the cardiovascular system (ATC C) (mean 2.61 ± 1.45), and the gastrointestinal tract and metabolism (ATC A) (mean 1.92 ± 1.54). Women consumed significantly more medications belonging to ATC N (3.37 ± 1.74) than men (1.86 ± 1.32) (U = 291.0, *p* < 0.001). There were also significant differences (<0.05) between the sexes in the consumption of ATC G and R drugs (Table 1). Among the most used drugs in ATC N, N06A antidepressants (mean 0.59 ± 0.69), N05B anxiolytics (mean 0.52 ± 0.70), and N05A antipsychotics (mean 0.39 ± 0.73) stand out. In ATC C, those with the highest average consumption were C10A lipid-modifying agents (mean 0.52 ± 0.53). Drugs for peptic ulcers and gastroesophageal reflux disease (ATC A02B) were the most consumed among ATC A drugs with a mean = 0.60 ± 0.49.

The pharmacotherapy complexity index was high (mean 19.53 ± 8.87) with statistically significant differences between sexes (U = 330.0; *p* = 0.005). Women had a higher mean complexity index (21.31 ± 9.24) than men (14.93 ± 5.82). The dosing frequency component (Subtotal B) contributed most to the total complexity score (11.70 ± 5.55) (Table 2).

Approximately 97% (n = 73) of users had at least one potential drug interaction, with a mean of 11.21 ± 9.60 interactions per user, ranging from 0 to 44 interactions. Type C interactions (monitor therapy) were the most prevalent (93.3%; n = 70) with a mean of 7.92 ± 7.23 interactions per user (Table 2); no statistically significant differences were found between sexes for interactions. Although not very common, type D and type X interactions are those that require closer monitoring. Table 3 shows the drug pairs classified as type D and X interactions, describing the reason for the interaction. Among the potential type X drug interactions, the following stand out: Clopidogrel + Omeprazole; Cholecalciferol + Paricalcitol; Tiapride + Carbidopa/Levodopa; Amisulpride + Carbidopa/Levodopa; Azelastine Nasal + CNS depressants (Alprazolam, Gabapentin, Trazodone).

Regarding PIMs, 86.7% (n = 65) of users were prescribed at least one potentially inappropriate medication, with a mean of 2.04 ± 1.41 PIMs per user. The distribution of PIMs differed significantly between sexes (U = 381.5; *p* = 0.025) (Table 2). The most frequently identified PIMs belonged to ATC N (mean 0.89 ± 0.86), specifically anxiolytics (ATC N05B) (mean 0.39 ± 0.59), followed by ATC A (mean 0.76 ± 0.68), primarily drugs for peptic ulcer treatment (A02B) (mean 0.59 ± 0.55).

### Anticholinergic Burden

The anticholinergic burden was calculated using two different tools: the ACB calculator and CALS—CRIDECO. Using the ACB calculator, 90.7% (n = 68) of users had a pharmacotherapeutic profile with anticholinergic burden, with a mean burden of 3.60 ± 2.84. Approximately 59% (n = 44) of users had an anticholinergic burden ≥ 3. Using the CALS—CRIDECO tool, 89.3% (n = 67) of users showed an anticholinergic load, with a mean value of 3.33 ± 2.51. It should be noted that these high prevalence figures reflect the classification of any score > 0 as indicative of anticholinergic burden; the clinically more meaningful threshold of a score ≥ 3, associated with increased adverse outcomes in geriatric populations, was present in approximately 59% of participants. The Wilcoxon test showed no statistically significant differences between the medians of the scores obtained by the two scales (*W* = 306.0; *p* = 0.055). A very strong, statistically significant positive correlation was found between the two tools (ρ = 0.896; *p* = 0.001), as well as substantial agreement as indicated by Cohen’s weighted Kappa coefficient (*k* = 0.73; 95% CI: 0.657–0.803; *p* < 0.001).

**Figure 1:**
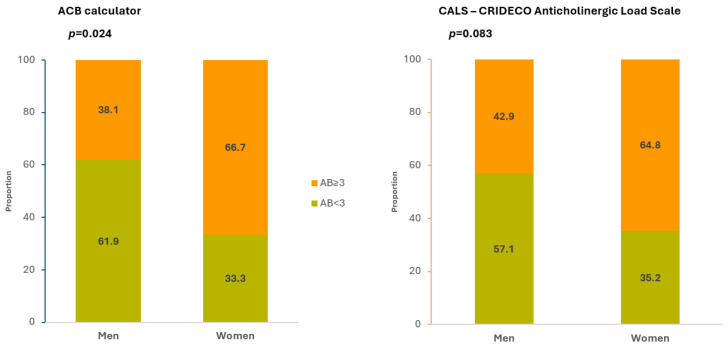
Comparison of relevant anticholinergic burden on the ABC calculator and CALS—CRIDECO scales between sexes. AB: Anticholinergic Burden.

The anticholinergic burden was significantly higher for women than for men in both tools (*p* < 0.05) (Table 2). However, when we analyzed the anticholinergic burden considered “relevant” (score ≥ 3), the tools yielded slightly different results regarding sex differences. The ACB calculator identified 8 men (38.1%) and 36 women (66.7%) with a relevant anticholinergic burden, indicating a significant difference between sexes (*p* = 0.024). Conversely, CALS—CRIDECO identified 9 men (42.9%) and 35 women (64.8%) with a relevant burden, but this difference was not statistically significant (*p* = 0.083) (Figure 1).

As expected, the anticholinergic burden varies significantly depending on the degree of polypharmacy for both the ACB (*p* = 0.020) and CALS—CRIDECO (*p* = 0.022). *Post hoc* analysis revealed that differences occurred specifically between the “polypharmacy” and “excessive polypharmacy” groups for both tools (ACB: *p* = 0.031; CALS—CRIDECO: *p* = 0.034). Thus, patients with excessive polypharmacy had a significantly higher anticholinergic burden than those with standard polypharmacy. A moderate and significant correlation was found between the total number of medications and the ACB score (ρ = 0.499; *p* < 0.001), as well as between the total number of medications and the CALS—CRIDECO score (ρ = 0.508; *p* < 0.001).

Correlations were also identified between the number of drugs in specific ATC groups and anticholinergic burden. Specifically, moderate correlations were found between the number of ATC N drugs and the burden calculated by ACB (ρ = 0.663; *p* < 0.001) and CALS—CRIDECO (ρ = 0.577; *p* < 0.001). Antipsychotics (N05A), anxiolytics (N05B), and antidepressants (N06A) showed significant associations with both anticholinergic scales (*p* < 0.05).

A weak but statistically significant positive correlation was observed between the number of potential drug–drug interactions and anticholinergic burden for both ACB (ρ = 0.262; *p* = 0.023) and CALS—CRIDECO (ρ = 0.258; *p* = 0.025). Users without potential interactions presented no anticholinergic burden. However, those with identified interactions had a mean burden of 3.70 ± 2.82 (ACB) and 3.42 ± 2.48 (CALS—CRIDECO), representing a statistically significant difference compared to those without interactions (U_ACB_ = 141.0, *p*_ACB_ = 0.009; U_CALS_ = 140.0, *p*_CALS_ = 0.012).

Anticholinergic burden also varied according to the complexity of pharmacotherapy (MRCI), showing a moderate and statistically significant correlation for both ACB (ρ = 0.458; *p* < 0.001) and CALS (ρ = 0.457; *p* < 0.001). The Mann–Whitney test indicated that the anticholinergic burden calculated by ACB differed significantly between users with low and high pharmacotherapy complexity (U = 962.5, *p* = 0.003); similar results were found for CALS—CRIDECO (U = 926.0, *p* = 0.010).

Finally, regarding PIMs, users with at least one prescribed PIM had a mean anticholinergic burden of 3.92 ± 2.83 (ACB) and 3.65 ± 2.48 (CALS—CRIDECO). In contrast, users with no PIMs had a lower mean burden (ACB: 1.50 ± 2.01; CALS—CRIDECO: 1.30 ± 1.70). A weak but statistically significant positive correlation was found between PIMs and burden for both ACB (ρ = 0.319; *p* = 0.005) and CALS—CRIDECO (ρ = 0.346; *p* = 0.002).

## 4. Discussion

This study evaluated the anticholinergic burden in the pharmacotherapeutic profiles of institutionalized elderly people using two tools and analyzed its relationship with several pharmacotherapeutic variables. The sociodemographic characteristics of the sample show a very aged population (M = 85.62 ± 7.62 years) consisting mostly of women (72%; n = 54). Portugal currently has the second highest aging index in Europe [38]. Data from 2024 show an aging index of 192.4%, with average life expectancy higher for women (84 years) than for men (78.7 years) [39] Other studies conducted on institutionalized older adults in Portugal and other European countries show similar sociodemographic characteristics [40,41,42].

The present study revealed a substantial anticholinergic burden among institutionalized elderly patients, with over 90% of participants exposed to medications with anticholinergic properties. This finding is particularly concerning given that approximately 59% of users presented a clinically relevant anticholinergic burden (score ≥ 3), a threshold associated with increased risk of cognitive impairment, falls, and mortality in geriatric populations [11,43,44,45]. The mean anticholinergic burden scores (ACB: 3.60 ± 2.84; CALS—CRIDECO: 3.33 ± 2.51) observed in this study were higher than those found in another recent study, although the percentage of patients with high anticholinergic burden was similar (CALS—CRIDECO: 58.7%) [46], underscoring the pervasiveness of this issue in vulnerable elderly populations.

The strong positive correlation (ρ = 0.896; *p* = 0.001) and substantial agreement (Cohen’s weighted Kappa = 0.730; 95% CI: 0.657–0.803) between the ACB and CALS—CRIDECO tools validate the consistency of anticholinergic burden assessment in this population. The absence of statistically significant differences between the median scores of the two scales (*p* = 0.055) further supports their comparability. Although these tools are often used to compare individuals with and without cognitive impairment, other studies show similar concordance results [31,47]. It should be noted, however, that the strong agreement between the two tools partly reflects their shared underlying construct: both instruments assess exposure to drugs with known anticholinergic activity using partially overlapping drug lists and analogous scoring logic. The observed agreement should therefore be interpreted as evidence of consistency between two complementary instruments rather than as independent validation of either scale.

A notable finding was the significantly higher anticholinergic burden observed in women compared to men across both assessment tools (ACB: *p* = 0.002; CALS—CRIDECO: *p* = 0.031), as observed by Gromek et al. [48]. Women demonstrated mean ACB scores of 4.19 ± 2.95 versus 2.10 ± 1.87 in men. This gender disparity may be explained by several interconnected factors identified in our results. First, women consumed significantly more medications from the ATC N (nervous system) category (3.37 ± 1.74 vs. 1.86 ± 1.32; *p* < 0.001), which includes drug classes with high anticholinergic properties such as antipsychotics, anxiolytics, and antidepressants. The higher prevalence of neuropsychiatric conditions in elderly women, including depression and anxiety disorders, may drive this increased prescription pattern [49,50]. Additionally, women in this cohort exhibited higher medication regimen complexity (MRCI: 21.31 ± 9.24 vs. 14.93 ± 5.82; *p* = 0.005) and consumed more potentially inappropriate medications (2.26 ± 1.40 vs. 1.48 ± 1.33; *p* = 0.025), suggesting a more complex pharmacotherapeutic profile that inherently carries greater anticholinergic risk. It should be acknowledged, however, that MRCI scores are not fully independent of medication count, as a higher number of medications inherently contributes to greater regimen complexity. The higher MRCI observed in women is therefore at least partially attributable to their greater number of prescribed medications, and the two measures should not be treated as entirely distinct. Nevertheless, MRCI captures additional dimensions that provide complementary information beyond a simple medication count.

Interestingly, while both tools identified higher anticholinergic burden in women, only the ACB calculator revealed statistically significant differences when analyzing clinically relevant burden (≥3) between sexes (*p* = 0.024), whereas CALS—CRIDECO did not (*p* = 0.083).

This discrepancy highlights the importance of tool selection in anticholinergic burden assessment and suggests that different scales may have varying sensitivity in detecting gender-specific differences, possibly due to differences in drug classification or scoring methodologies [47,51]. These differences likely reflect differences in the drug lists and scoring systems of the two scales rather than a definitive biological or clinical effect [18,47,51,52]. Clinicians should be aware of these nuances when selecting assessment tools and interpreting results, particularly in research contexts where consistent methodology is critical. Also, it should be acknowledged that these sex differences are unadjusted for potential confounders such as underlying diagnoses, cognitive status, functional level, and severity of comorbidities, which may differ systematically between men and women in nursing homes. As such, the observed differences may partly reflect residual confounding rather than a direct sex effect on prescribing patterns.

The study demonstrated a clear association between polypharmacy and anticholinergic burden. Patients with excessive polypharmacy (≥10 medications) exhibited significantly higher anticholinergic burden compared to those with standard polypharmacy (5–9 medications) for both ACB (*p* = 0.031) and CALS—CRIDECO (*p* = 0.034). The moderate positive correlations between total medication count and anticholinergic burden (ACB: ρ = 0.499, *p* < 0.001; CALS—CRIDECO: ρ = 0.508, *p* < 0.001) suggest a relationship where each additional medication incrementally increases the probability of anticholinergic exposure [44,53,54,55]. This relationship is particularly concerning given that 56% of the study population was classified as excessively polymedicated, a result consistent to previous studies [27]. The cumulative anticholinergic effect of multiple medications, even those with individually low anticholinergic activity, can result in clinically significant burden [56]. This phenomenon underscores the importance of comprehensive medication review in institutionalized older adults, particularly focusing on deprescribing strategies to reduce both overall medication burden and associated anticholinergic load [57,58].

The consumption of ATC N drugs in institutionalized elderly people is high [4,59,60], as shown in this study. The moderate correlation between ATC N (nervous system) medications and anticholinergic burden (ACB: ρ = 0.663, *p* < 0.001; CALS—CRIDECO: ρ = 0.577, *p* < 0.001) identifies this therapeutic category as the primary contributor to anticholinergic exposure in this population. Specifically, antipsychotics (N05A), anxiolytics (N05B), and antidepressants (N06A) demonstrated significant associations with both anticholinergic scales (*p* < 0.05). In fact, antidepressants, antipsychotics, and anxiolytics are the most common drugs on various anticholinergic burden lists. Substances such as amitriptyline, imipramine, or paroxetine as antidepressants, diazepam as anxiolytics, or olanzapine as antipsychotics are examples of drugs that contribute to anticholinergic burden in many anticholinergic burden assessment tools [61]. This finding has important clinical implications. While psychotropic medications are often necessary for managing behavioral and psychological symptoms in institutionalized elderly, clinicians should prioritize agents with minimal anticholinergic properties within these classes [62,63]. For instance, selective serotonin reuptake inhibitors (SSRIs) generally have lower anticholinergic activity compared to tricyclic antidepressants. Furthermore, clinicians should consider deprescribing benzodiazepines where appropriate [64,65,66]. The high consumption of nervous system medications (mean: 2.95 ± 1.76 drugs per user) suggests substantial opportunity for therapeutic optimization through substitution with lower anticholinergic alternatives.

The moderate positive correlation between medication regimen complexity index (MRCI) and anticholinergic burden (ACB: ρ = 0.458, *p* < 0.001; CALS—CRIDECO: ρ = 0.457, *p* < 0.001) reveals an important relationship between treatment complexity and anticholinergic exposure. Users with high pharmacotherapy complexity demonstrated significantly higher anticholinergic burden compared to those with low complexity (ACB: *p* = 0.003; CALS—CRIDECO: *p* = 0.010). Complex pharmacotherapeutic regimens are common in institutionalized older adults, primarily driven by polypharmacy [4,67,68]. The high number of medications administered to older adults, associated with high comorbidity, increases the likelihood of incorporating drugs with anticholinergic properties [69]. The relationship between therapeutic complexity and anticholinergic burden suggests that efforts to simplify medication regimens, through once-daily formulations or combination products, but mainly through deprescribing, may simultaneously reduce anticholinergic exposure, although further studies on the effectiveness of this type of intervention are needed [70,71,72].

Although the correlation between potential drug–drug interactions (PDDI) and anticholinergic burden was weak (ACB: ρ = 0.262, *p* = 0.023; CALS—CRIDECO: ρ = 0.258, *p* = 0.025), it was statistically significant and clinically relevant. Notably, all users without PDDI also had no anticholinergic burden, while those with identified interactions exhibited mean burdens of 3.70 ± 2.82 (ACB) and 3.42 ± 2.48 (CALS—CRIDECO), with statistically significant differences (*p* < 0.01). This relationship likely reflects the fact that both drug interactions [73,74] and anticholinergic burden [9,75,76], like the medication regimen complexity [67,68,77], are consequences of polypharmacy. Patients taking multiple medications are simultaneously at higher risk for drug interactions and for accumulating anticholinergic effects [73]. In addition, certain combinations of drugs can produce additive or synergistic anticholinergic effects, even when the individual agents have modest anticholinergic properties, highlighting the importance of calculating the anticholinergic load in older adults [78,79].

The positive correlation between potentially inappropriate medications (PIMs) and anticholinergic burden (ACB: ρ = 0.319, *p* = 0.005; CALS—CRIDECO: ρ = 0.346, *p* = 0.002) was expected, as many PIMs in elderly populations are inappropriate precisely due to their anticholinergic properties [80,81,82,83]. Users with at least one PIM exhibited substantially higher anticholinergic burden (ACB: 3.92 ± 2.83; CALS—CRIDECO: 3.65 ± 2.48) compared to those without PIMs (ACB: 1.50 ± 2.01; CALS—CRIDECO: 1.30 ± 1.70). The most consumed PIMs belonged to ATC N (nervous system; mean 0.89 ± 0.86), particularly anxiolytics (mean 0.39 ± 0.59). The high consumption of anxiolytics, many of which have anticholinergic effects, directly contributes to the overall anticholinergic burden [84,85,86]. The substantial overlap between PIMs and anticholinergic burden (87% of users had at least one PIM; 90.7% had anticholinergic burden) suggests that interventions aimed at reducing PIMs could significantly decrease anticholinergic exposure in this population [87]. The implementation of deprescribing protocols, with specific attention to anticholinergic properties, may bring dual benefits in reducing both inappropriate prescribing and adverse outcomes related to anticholinergics [75,88,89]. However, some barriers associated with the assessment of anticholinergic burden and the deprescribing of medications still need to be overcome [88,90,91].

### 4.1. Limitations

This study has several limitations that must be acknowledged. First, the cross-sectional design allows for the identification of associations between variables (such as polypharmacy and anticholinergic burden) but prevents the establishment of temporal or causal relationships. Second, the final sample size (n = 75) was smaller than the initially estimated target of 150 participants. The original calculation aimed for a margin of error of 5% at the 95% confidence level; the achieved sample yields a margin of error of approximately 10%, which, while acceptable for a descriptive study, may limit the statistical power of some sub-group analyses (e.g., sex-stratified comparisons). This constraint was due to practical difficulties in obtaining informed consent and incomplete pharmacotherapeutic profiles in a proportion of eligible patients. Furthermore, as the participants were recruited via a single community pharmacy in the Alentejo region, the findings may reflect specific local prescribing patterns and may not be fully generalizable to the broader population of institutionalized older adults in Portugal or other European regions.

Third, the type of medication review that was conducted does not allow us to verify medication adherence (whether the dispensed medication was actually consumed) nor to correlate the calculated anticholinergic burden with actual clinical outcomes, such as the incidence of falls, delirium, or clinically diagnosed cognitive decline in this specific cohort. Furthermore, the absence of clinical data on comorbidities, cognitive status, and functional level prevents adjustment for these potential confounders, limiting the interpretability of the observed associations, particularly those related to sex differences and polypharmacy.

The exclusion of patients taking only one medication may influence the results of the anticholinergic load, as this medication could have anticholinergic properties. However, the analysis of potential drug interactions led us to include only elderly patients taking two or more medications.

### 4.2. Clinical Implications and Future Directions

The findings of this study have several important clinical implications for the care of institutionalized elderly patients. The high prevalence of clinically relevant anticholinergic burden demonstrates the need for periodic multidisciplinary medication reviews, mainly because the association between anticholinergic burden and modifiable factors (polypharmacy, medication complexity, PIMs) identifies clear targets for intervention. The gender differences observed in this study deserve special attention. Women in care facilities may require more intensive monitoring of anticholinergic adverse effects and may benefit from specific prescription reduction interventions. Considering that women have a higher average life expectancy, we believe that further research is needed to elucidate the mechanisms underlying these gender differences and to develop gender-specific prescribing guidelines. These suggestions should be regarded as hypotheses for future research rather than evidence-based clinical guidelines, given the cross-sectional and unadjusted nature of the current findings. To date, gender-specific prescribing protocols for anticholinergic management in nursing homes remain an understudied area warranting dedicated prospective investigation. More intervention work should be developed in terms of medication review with multidisciplinary teams (doctors, pharmacists, nurses), with clear protocols that can be implemented in nursing homes.

## 5. Conclusions

This study demonstrates that anticholinergic burden is highly prevalent among institutionalized elderly patients and is significantly associated with polypharmacy, medication regimen complexity, potentially inappropriate medications, and consumption of nervous system medications. Women are particularly vulnerable to high anticholinergic burden, an association driven by their greater consumption of psychotropic medications and higher overall medication complexity. These findings highlight the rationale for systematic assessment of anticholinergic burden in long-term care settings, and identify potential targets for future intervention, including deprescribing, therapeutic substitution, and regimen simplification that warrant evaluation in prospective studies.

## Figures and Tables

**Table 1:** Distribution of medication consumption by ATC (1st level).

ATC Code (M ± SD)	Total (n = 75)	Women (n = 54)	Men (n = 21)	*p* (Mann–Whitney Test)
No ATC	0.13 ± 0.34	0.13 ± 0.34	0.14 ± 0.35	0.881
ATC A	1.92 ± 1.54	1.98 ± 1.62	1.76 ± 1.34	0.702
ATC B	1.03 ± 0.82	1.04 ± 0.87	1.00 ± 0.71	0.979
ATC C	2.61 ± 1.45	2.52 ± 1.54	2.86 ± 1.20	0.238
ATC D	0.01 ± 0.12	0.00 ± 0.00	0.05 ± 0.22	0.109
ATC G	0.21 ± 0.47	0.13 ± 0.44	0.43 ± 0.51	0.002
ATC H	0.13 ± 0.48	0.13 ± 0.48	0.14 ± 0.48	0.953
ATC L	0.04 ± 0.20	0.04 ± 0.19	0.05 ± 0.22	0.835
ATC M	0.33 ± 0.53	0.31 ± 0.54	0.38 ± 0.50	0.458
ATC N	2.95 ± 1.76	3.37 ± 1.74	1.86 ± 1.32	<0.001
ATC R	0.20 ± 0.52	0.28 ± 0.60	0.00 ± 0.00	0.027
ATC S	0.19 ± 0.59	0.24 ± 0.67	0.05 ± 0.22	0.171

**Table 2:** Distribution of pharmacotherapeutic variables.

	Total (n = 75)	Women (n = 54)	Men (n = 21)	*p* (Mann–Whitney Test)
MRCI	19.53 ± 8.87	21.31 ± 9.24	14.93 ± 5.82	0.005
Subtotal A	2.36 ± 2.14	2.59 ± 2.34	1.76 ± 1.38	0.139
Subtotal B	11.70 ± 5.55	12.70 ± 5.72	9.12 ± 4.15	0.016
Subtotal C	5.47 ± 3.12	4.05 ± 2.25	6.02 ± 3.25	0.012
PDDI (Total)	11.21 ± 9.60	12.17 ± 10.31	8.76 ± 7.08	0.202
Type A Interactions	0.08 ± 0.59	0.11 ± 0.48	0.00 ± 0.00	0.375
Type B Interactions	1.91 ± 3.43	2.30 ± 3.92	0.90 ± 1.22	0.037
Type C Interactions	7.92 ± 7.23	8.41 ± 7.49	6.67 ± 6.51	0.369
Type D Interactions	1.19 ± 2.14	1.19 ± 2.18	1.19 ± 2.06	0.792
Type X Interactions	0.12 ± 0.43	0.03 ± 0.51	0.00 ± 0.00	0.085
Potentially inappropriate medications	2.04 ± 1.41	2.26 ± 1.40	1.48 ± 1.33	0.025
Anticholinergic Burden (ACB)	3.60 ± 2.84	4.19 ± 2.95	2.10 ± 1.87	0.002
Anticholinergic Burden (CALS—CRIDECO)	3.33 ± 2.51	3.72 ± 2.64	2.33 ± 1.85	0.031

MRCI—Medication Regimen Complexity; PDDI—Potential Drug–Drug Interactions.

**Table 3:** Description of the potential drug-drug interactions (C, D and X) identified.

Interaction Type	System/Group	Risk/Mechanism	Specific Drug Combinations
Type C	Hematology & Antiplatelet Therapy	Interactions affecting the efficacy of Clopidogrel or increasing bleeding risks.	Clopidogrel + Pantoprazol */Sertraline */Trazodone
	Central Nervous System (CNS)	Risk of Serotonin Syndrome, excessive sedation, or increased risk of seizures.	Escitalopram */Sertraline + Tramadol *
			Quetiapine * + Sertraline */Trazodone
			Alprazolam * + Lorazepam *
			Escitalopram */Omeprazole
			Tramadol * + Pregabalin
	Endocrine and Metabolic System	Combined use increases the risk of myopathy and rhabdomyolysis	Fenofibrate + Ezetimibe/Rosuvastatin/Pravastatin
	Cardiovascular & Renal System	Interactions affecting renal function, blood pressure, or risk of myopathy.	Olmesartan/Amlodipine/Hydrochlorothiazide + Escotalopram */Tramadol */Metformin */Colecalciferol
			Furosemide + Allopurinol/Quetiapine * Candesartan/Hydrochlorothiazide
			Allopurinol + Hydrochlorothiazide
Type D	Endocrine System & Diabetes	Risk of hypoglycemia or metabolic changes when combining insulins/sulfonylureas with other agents.	Degludec + Dapagliflozin
			Glulisine + Dapagliflozin
			Glargine + Empagliflozin
			Degludec + Empagliflozin
			Degludec/Gluisine + Dulaglutide
			Lispro/Glargine/Degludec + Linagliptin/Vildagliptin
			Gliclazide + Dapagliflozin/Dulaglutide
	Central Nervous System (CNS)	Risk of falls and respiratory depression due to additive CNS depressant effects.	Zolpidem + Midazolam */Mirtazapine */Levetiracetam/Pregabalin/Valproic Acid */Hydroxyzine */Alprazolam *
			Clozapine * + Carbamazepine/Hydroxyzin */Lorazepam *
			Haloperidol */Escitalopram * + Quetiapine *
			Acetaminophen/Tramadol * + Alprazolam */Diazepam */Lorazepam */Bromazepam
			Tramadol * + Pregabalin
	Parkinson’s Disease & Dopaminergic Interactions	Interactions that may reduce treatment efficacy or exacerbate psychotic symptoms.	(Carbidopa + Levodopa) * + Quetiapine */Risperidone *
			Carbidopa/Levodopa * + Ferrous Sulfate *
	Cardiovascular & Renal System	Interactions affecting renal function, blood pressure, or risk of myopathy.	Simvastatin + Amlodipine/Candesartan
			Rosuvastatin + Febuxostat
			Furosemide + Celecoxib */Etoricoxib */Clonixin/Tramadol * + Dexketoprofen
			Bisoprolol + Rilmenidine
			Nebivolol + Rivastigmine
	Other Interactions	Hemorrhagic/gastric risk, absorption issues, or immunosuppression.	Aspirin + Aceclofenac/Ibuprofen/Metamizole/Ginkgo Biloba
			Naproxen + Escitalopram *
			Levothyroxine + Folic Acid/Ferrous Sulfate/Calcium Carbonate/Colecalciferol
			Methotrexate + Clopidogrel + Aspirin or Omeprazole *
Type X	Gastrointestinal/Hematologic	Decreased efficacy of Clopidogrel; avoid combination.	Clopidogrel + Omeprazole *
	Vitamin Toxicity	Risk of toxicity from multiple Vitamin D analogs.	Colecalciferol + Paricalcitol.
	Parkinson’s/Antipsychotics	Combinations listed as contraindicated in product labeling.	Tiapride + Carbidopa/Levodopa *
			Amisulpride + Anti-Parkinson agents (Dopamine agonists).
	Respiratory/CNS	Potential for enhanced CNS depressant effects; avoid combination.	Nasal Azelastine * + CNS Depressants (Alprazolam *, Gabapentin, Trazodone).

* anticholinergic drugs.

## Data Availability

Due to privacy restrictions, the data presented in this study are only available on request from the corresponding author.

## Abbreviations

The following abbreviations are used in this manuscript:

ACB	Anticholinergic Cognitive Burden
ATC	Anatomical Therapeutic Chemical
CALS—CRIDECO	CRIDECO Anticholinergic Load Scale
GABS	German Anticholinergic Burden Scale
MRCI	Medication Regimen Complexity Index
PCNE	Pharmaceutical Care Network Europe
PIM	Potentially Inappropriate Medications
PDDI	Potential Drug–Drug Interactions
